# Human trophoblast function during the implantation process

**DOI:** 10.1186/1477-7827-3-56

**Published:** 2005-10-20

**Authors:** Elsebeth Staun-Ram, Eliezer Shalev

**Affiliations:** 1 Laboratory for Research in Reproductive Sciences, Department of Obstetrics and Gynecology, Ha'Emek Medical Center, 18101, Afula, Israel; 2 Rappaport Faculty of Medicine, Technion-Israel Institute of Technology, Haifa, Israel

## Abstract

The implantation process involves complex and synchronized molecular and cellular events between the uterus and the implanting embryo. These events are regulated by paracrine and autocrine factors. Trophoblast invasion and migration through the uterine wall is mediated by molecular and cellular interactions, controlled by the trophoblast and the maternal microenvironment. This review is focused on the molecular constituents of the human trophoblast, their actions and interactions, including interrelations with the uterine endometrium.

## 1. Introduction

**Figure 1:**
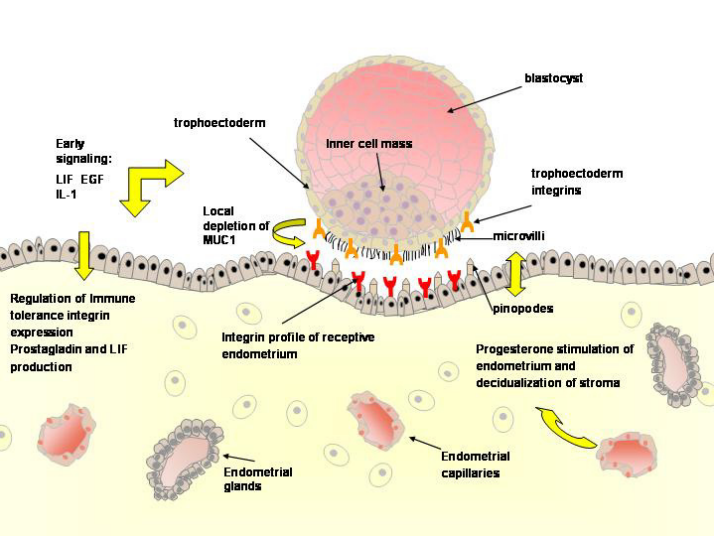
A schematic representation of a blastocyst approaching the receptive endometrium, defined by the integrin profile and appearance of pinopodes. Early signaling between the blastocyst and the endometrium precedes the attachment.

**Figure 2:**
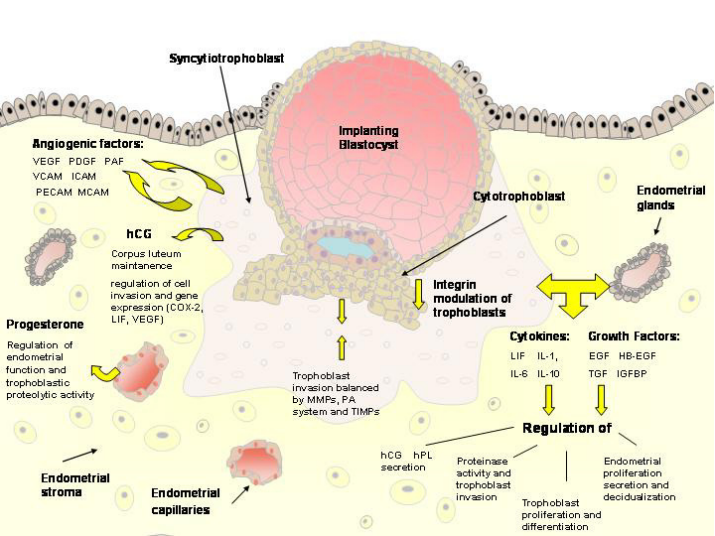
A schematic representation of an implanting blastocyst, highlighting interactions between trophoblastic and endometrial cells, including integrins, growth factors, cytokines, hormones and proteases.

Successful implantation depends on synchronization between the developmental stages of the embryo itself and the complex series of molecular and cellular events that are induced in the pregnant uterus by paracrine and autocrine regulators [1]. Embryos prepare for implantation during their cleavage stage. Successive cleavages must produce sufficient cells by the time the blastocyst is formed to permit the full formation of inner cell mass and trophectodermal cells. The latter are the origin of the cytotrophoblastic cells which ensue to become either the villous cytotrophoblastic cells which will proliferate and differentiate by fusion to form the syncytiotrophoblast, or they will stream out of the syncytiotrophoblast to form mononuclear multilayered invasive extravillous cytotrophoblastic cells [2]. The process of implantation begins six to seven days following fertilization [3] and consists basically of three stages [4]. Apposition is the first stage denoting the initial, still unstable, adhesion of the blastocyst to the uterine wall. At this stage the pinopodes, which are micro protrusions from the apical uterine epithelium surface, inter-digitate with microvilli on the apical syncytiotrophoblast surface of the blastocyst[5] (Figure 1). The stable adhesion, which is the next step, reveals an increased physical contact between the blastocyst and the uterine epithelium, while the embryonic pole is oriented toward the epithelium. The last stage is the invasion process, which starts with the penetration of the syncytiotrophoblasts through the uterine epithelium and followed by infiltration of the mononuclear cytotrophoblasts invading the entire endometrium, the inner third of the myometrium and the uterine vasculature. Villous cytotrophoblast cells at the tip of the anchoring villi proliferate outwards from the underlying basement membrane to form cell columns from which cells migrate into the decidua (interstitial trophoblast cells) and invade the maternal spiral arteries and designate the endovascular trophoblast cells. The latter allows the trophoblasts to be in direct contact with the maternal blood establishing the uteroplacental circulation [4]. Trophoblast invasion and migration is probably controlled by components of the trophoblast itself and maternal microenvironment, through molecular and cellular interaction (Figure 2).

The human implantation process is unique thus no other mammals can provide a true model [2]. Ethical restrictions and limited availability of human placental tissue limits the possibilities of human implantation studies. Consequently most knowledge comes from *in vitro *experiments using cultured human trophoblasts or cell-lines, mainly obtained from choriocarcinoma. Although the differences in reproductive physiology between species limit the relevance, knowledge on the possible role of various factors in the implantation process comes from knockout-studies in mice. Primates provide a more physiological relevant model, therefore *in vivo *and *in vitro *studies in baboons have contributed to our knowledge [6]. All in all, human *in vitro *and mammal *in vitro *and *in vivo *studies have provided important information, which helped in understand at least part from the complex process of implantation. This review focuses on the molecular constituents of the human trophoblast, their actions and interactions, which seem to be crucial for successful implantation.

## 2. Cell adhesion molecules

### 2.1 Integrins

During the mid-luteal stage of the menstrual cycle, apparently in response to progesterone, epithelial apical membrane projections named pinopodes appear in the uterus [5]. The biological nature of the pinopodes has not been determined, but their short appearance during the implantation window, interconnecting with microvilli on the apical syncytiotrophoblast surface of the blastocyst, suggests an involvement in embryo implantation [7]. Adherence of the trophectoderm cells of the blastocyst to the pinopode membrane has been suggested to occur through adhesive molecules such as E-cadherin, present in the pinopode epithelial membrane [5]. *In vitro *studies showed that although no direct contact between trophectoderm and pinopode is seen, blastocysts tends to attach to areas of cultured endometrial epithelium containing pinopodes [5].

Integrins are heterodimeric membrane glycoproteins, composed of α and β subunits, capable of binding to various extracellular matrix (ECM) components and cell adhesion molecules, thereby influencing and mediating adhesion, migration, invasion, cytoskeleton reorganization and cellular signaling [8]. Endometrial integrins are hormone-dependent and vary throughout the menstrual cycle [9]. The integrin repertoire of the endometrium may play an important role for obtaining a successful implantation. According to a timed expression correlating with embryo attachment, the αVβ3 and the α4β1 integrins are considered markers of uterine receptivity [10-12]. αVβ3 has been shown to be highly expressed at the time of embryo attachment, and aberrant expression of αVβ3 is associated with infertility [13,14]. Women with recurrent miscarriages were found to have a lower concentration of α4β1 and α5β1 integrins in the stroma during the implantation window, than women with unexplained infertility [15]. The relevance of integrins alteration, in glandular epithelium or stroma, but not in luminal epithelium is not well understood, since they are not likely to be involved in the early parts of implantation [16]. The trophectoderm also express several integrins, α3, α5, β1, β3, β4 and β5, supposed to be implicated in blastocyst attachment to the endothelial surface [17-19]. Trophoblasts modulate their integrin repertoire during invasion of the stroma and during differentiation, varying in invasive and non-invasive cells [2,16]. Several factors known to be involved in the implantation process may act through mediation of integrins. For example, Insulin-like growth factor (IGF)-I-induced migration of extravillous trophoblast cells (EVT) probably involves internalization of the α5β1-integrin on EVT [19], as well as influencing the αVβ3-integrin pathway [18]. In female mice lacking a functional integrin β1 gene, embryos develop normally to the blastocyst stage but fail to implant properly and die [20]. No other integrins were found in knockout studies to be involved in implantation defects [21], but inactivation of αVβ3 by the disintegrin echistatin significantly reduced implantation sites in mice [22]. Apparently several integrins may have a redundant role in implantation [23].

### 2.2 MUC1

Uterine cell-surface glycoproteins such as MUC1 are thought to provide a barrier to trophoblast invasiveness, by controlling accessibility of integrin receptors to their ligands [8]. MUC1 increases from the proliferative to the secretory phase in endometrial tissue and then decreases in the late secretory phase [24]. Progesterone combined with estrogen up-regulates MUC1 at the receptive endometrium [24]. Blastocysts were shown to deplete MUC1 locally from the implantation site, whereas during the apposition phase, when the blastocyst apparently must be stationary in position, MUC1 was up-regulated [25]. MUC1 may thereby serve as an anti-adhesive molecule that hinders blastocyst attachment to the uterine epithelium until 3 days after entrance to the uterus [26].

## 3. Extracellular matrix proteins

The trophoblastic cells are confronted with various matrix proteins and basement membranes, when penetrating the uterine wall. These ECM components, including collagens (Col), fibronectin (FN), laminin (LN), vitronectin (VN), trophin and tastin, influence cell functions by binding to integrins, thereby effecting adhesion, migration, differentiation and spreading [2]. Trophin and Tastin for example can be found in endometrial epithelium as well as in trophoblasts, and may be involved in blastocyst attachment by forming a cell-adhesion molecular complex [26].

The components of the ECM are changed during the menstrual cycle [27]. The changes associated with decidualization, during the mid-secretory phase, are especially relevant for implantation. This includes an increase in hyaluronan, a decrease in collagen VI and a slower production of collagen III and I. During decidualization, stromal fibroblasts differentiate into decidual cells, and a basement membrane, containing laminin, entactin, collagen IV and heparan sulphate proteoglycan, appears around each cell [28].

ECM components affect the behavior and function of trophoblastic cells by affecting matrix metalloproteases (MMPs) and their tissue inhibitors (TIMPs) as shown by Xu et al, 2001 [29]. Trophoblasts, grown in the presence of VN, LN or FN, secreted more MMP-9 than in the presence of Col I, or IV, whereas MMP-2, TIMP-2 and MMP-14 were not affected. TIMP-3 on the contrary was inhibited by the presence of VN. Trophoblast adhesiveness was highest in the presence of Col I and IV compared with the other matrix proteins. Since cytotrophoblast cells also produce LN, FN and VN during the first trimester, these matrix proteins may be part of an autocrine mechanism, regulating MMP expression and cell invasiveness [29].

## 4. Growth factors

### 4.1 Epidermal Growth Factor

Epidermal Growth Factor (EGF) is expressed both in decidual and trophoblastic cells [30] and affects implantation in several ways. EGF induces trophoblast invasion [31,32], trophoblast differentiation [33,34] and trophoblast proliferation [35], and is therefore regarded a major regulator of the implantation process. EGF has been shown to increase MMP-2 and MMP-9 activity in trophoblastic cells [32,36,37], and also urokinase-type plasminogen activator (uPA) and plasminogen activator inhibitor-1 (PAI-1) activity [36] in trophoblastic cells, thereby inducing cell invasion. EGF stimulates secretion of human chorionic gonadotrophin (hCG) and human placental lactogen (hPL) from trophoblastic cells [38,33], and has been shown to induce α2 integrin expression in a choriocarcinoma cell-line, an effect found to be related to increased invasiveness [38].

### 4.2 Heparin-binding EGF-like growth factor

Heparin-binding EGF-like growth factor (HB-EGF) shares a common receptor with EGF and transforming growth factor (TGF)-α. HB-EGF is expressed in stromal and epithelial cells of the uterus and is thought to regulate endometrial proliferation, secretion and decidualization [21]. The expression of HB-EGF is maximal in the mid-secretory phase in uterine epithelial cells; therefore HB-EGF may also be involved in the regulation of blastocyst implantation [21].

### 4.3 Transforming growth factor β

TGF-β is expressed both in endometrial and trophoblastic cells [2]. TGF-β was shown to inhibit trophoblast proliferation and invasion apparently by stimulating TIMP secretion and decreasing MMP activation through down-regulation of plasminogen activators [39]. In another study TGF-β was found to inhibit trophoblast invasion by reducing MMP-9 and uPA secretion, but did not affect TIMP levels or cell proliferation [40]. Elevated TGF-β activity has been reported in the plasma of pre-eclamptic mothers [41] and may be implicated in the impaired implantation associated with pre-eclampsia [42].

### 4.4 Insulin-like growth factor binding protein-1

Insulin-like growth factor binding protein-1 (IGFBP-1) is the main secretory product of the decidualized endometrium. IGFBP-1 modulates the metabolic effect of insulin-growth factor (IGF)-I and IGF-II and has been shown to increase the gelatinolytic activity of trophopblasts [43] and trophoblast invasiveness mainly by increasing cell migration [44,45].

## 5. Cytokines

### 5.1 Leukaemia inhibitory factor

Leukaemia inhibitory factor (LIF) secreted from the uterus is regarded an important factor in embryo implantation. Female mice lacking a functional LIF gene are fertile, but their blastocysts fail to implant, even though they are viable and can implant when transferred to wild-type recipients [46]. Maximal expression of LIF in endometrial epithelium is during the implantation window [21]. LIF and its receptor are also expressed in pre-implantation embryos [47] and in cytotrophoblasts [48]. LIF inhibits gelatinase activity in cytotrophoblasts, thereby effecting cell invasiveness [49]. LIF may also be involved in immune tolerance through regulation of HLA-G, a class I MHC molecule specifically expressed by invasive cytotrophoblast cells [50].

### 5.2 Interleukin-1

Interleukin-1 (IL-1) is present at the feto-maternal interphase; trophoblastic cells and decidualized stromal cells produce IL-1, and the IL-1 receptor is present in endometrial epithelial cells as well as in trophoblasts [2]. IL-1 may be one of the first signals of the blastocyst acting upon the endometrium, since *in vitro *IL-1 increases endometrial secretion of prostaglandin E_2_, LIF and of integrin β_3 _subunit expression [51]. In mice IL-1 receptor antagonist given prior to implantation significantly reduces the number of implanted embryos, indicating a role for IL-1 in embryo implantation [52]. IL-1 can stimulate MMP-9 activity in trophoblasts [53] and expression in endometrial stroma cells [54], thereby inducing trophoblast invasion Among other cytokines also present at the maternal-feto interface are IL-6, which stimulates MMP-2 and MMP-9 activity [55] whereas IL-10 down-regulates MMP-9 and trophoblast invasion [56].

## 6. Hormones

### 6.1 Human Chorionic Gonadotropin

Syncytiotrophoblastic cells secrete hormones including progesterone and human chorionic gonadotropin (hCG), which play important roles in the implantation process. hCG affects several processes during pregnancy, besides the well-known maintenance of the corpus luteum, including cell growth and differentiation. The trophoblasts themselves express a truncated and inactive hCG receptor until ninth week of gestation, then switching to the full length receptor, allowing hCG autocrinic regulation of various functions including cell differentiation in the trophoblasts [57]. In trophoblastic neoplasms these receptors have been found to be over-expressed [2]. Positive correlation between hCG level and trophoblast invasion has been shown in ectopic pregnancies [58,59], indicating a possible stimulatory effect on trophoblast invasiveness. hCG was found *in vitro *to increase invasiveness of a trophoblastic choriocarcinoma cell-line [60] however *in vitro *studies of primary trophoblast showed the opposite effect [61,62]. Therefore, the influence of hCG on trophoblast *in vivo *invasion remains unclear. hCG stimulates the cAMP pathway, and forskolin, which directly activates adenylate cyclase and increases cAMP, also stimulates trophoblast invasiveness, both in a trophoblast choriocarcinoma cell-line and in primary trophoblastic cells [32]. This is in agreement with the finding that hCG increases MMP-9, an important key-factor in trophoblast invasion [57]. Lately hCG was shown in vitro to stimulate trophoblast migration through an IGF-II effect [63].

hCG influences several uterine factors, for example increases the expression of COX-2 gene, an enzyme involved in prostaglandin biosynthesis [21], LIF and vascular endothelial growth factor (VEGF) [57], suggesting a role in endometrial vascularization. In the baboon hCG was shown to cause physiological effects on the uterine endometrium in vivo, including an increase in glycodelin expression and secretion by the glandular epithelium, and differentiation of subepithelial stromal fibroblasts characterized by expression of the alpha smooth muscle actin, associated with the initiation of decidualization [64,65]. This suggests that the primate blastocyst signal modulates the uterine environment prior to implantation [64].

Hyperglycosylated hCG (HhCG), also called invasive trophoblast antigen (ITA), is an hCG variant with extra-large O-linked oligosaccharides. HhCG is the predominant form of hCG in early pregnancy and in choriocarcinoma, where aggressive trophoblast invasion takes place [66]. It is produced by poorly differentiated or invasive trophoblast cells and decreases as pregnancy advances [67]. The fact that HhCG is dominant around the time of implantation and in the 3 weeks that follow [68] makes it an interesting molecule that deserves further exploration. Low maternal mid-trimester urine HhCG has been found to predict preeclampsia, which is associated with poor trophoblast invasion [69].

### 6.2 Progesterone

Progesterone has a crucial role in preparing the uterus for the developing embryo and for obtaining a successful pregnancy. Stromal cells differentiate into decidual cells in respond to progesterone during the decidualization process, characterized by morphological changes and secretion of prolactin [70]. Progesterone is also required for maintenance of the pregnancy by stimulating and maintaining uterine functions, necessary for early embryonic development, implantation, placentation and fetal development. Hormones such as hCG, produced by the trophoblast, maintain this progesterone production in early pregnancy [71]. Progesterone was suggested to affect trophoblast invasiveness through the down-regulation of MMP-9 [72]. Progesterone is known to restrain endometrial breakdown by inhibiting MMPs. This inhibitory effect implies that progesterone impedes the invasion of trophoblast cells into the endometrial tissue. In a recent report progesterone was found to decrease invasion and gelatinase expression in early first trimester trophoblast cells and to increase cell invasion and MMP-2 expression in late trophoblast cells [73]. A differential progesterone receptor (PR) profile was documented with the dominance of PRB in the early trophoblast and dominance of PRA in the late trophoblast [73]. This differential PR profile is compatible with the inverse temporal effect of the hormone on the trophoblast cells. In mice a similar dual role of progesterone in embryo implantation has been reported, when progesterone promoted attachment and invasion of primary trophoblasts, mainly through MMP-2 stimulation, but inhibited invasion of secondary trophoblasts [74].

## 7. Inflammatory factors

### 7.1 Cortico-releasing hormone

The implanting embryo suppresses the maternal immune process, thereby preventing rejection. Cortico-releasing hormone (CRH) is produced by both trophoblasts and placental deciduas [75]. In mice, implantation can be highly reduced by anti-CRH antibody, indicating a role in embryo implantation [76]. Fas and its ligand FasL, belongs to the tumor necrosis family (TNF). Fas and Fas-FasL interaction, plays an important role in the regulation of immune tolerance, mainly by inducing apoptosis in cells carrying Fas, including T and B lymphocytes [75]. FasL is expressed on cytotrophoblastc as well as on maternal decidual cells of the placenta [77]. CRH was found *in vitro *to stimulate FasL expression in extravillous human trophoblasts, thereby enabling them to induce apoptosis of the surrounding activated T lymphocytes [75]. Rats treated with a CRH receptor 1 (CRHR1) antagonist had diminished FasL endometrial expression and reduced number of implantation sites, suggesting that locally produced CRH promotes implantation and maintenance of early pregnancy mainly by killing activated T cells [75]. The CRH-Fas-FasL system is not the sole maternal immunetolerance mechanism preventing embryo rejection. Mice with an inactivating mutation of Fasl gene or CRH and CRHR1-deficient mice [78-80] can be fertile.

### 7.2 Tumor necrosis factor-α

The pleiotropic cytokine TNF-α and its two receptors are present in the endometrium, as well as in placental trophoblasts [81]. TNF-α was shown *in vitro *to increase uPA secretion from cytotrophoblasts, thereby properly enhances the degradation of fibronectin during trophoblast penetration of the endometrial ECM [84]. Although, TNF-α did not affect MMP-9 concentration, the up-regulation of uPA increases activation of MMP-9 through the plasminogen activation system, thereby enhancing trophoblast invasiveness [84]. This is consistent with another report describing TNF-α stimulation of MMP-9 gelatinolytic activity, without affecting MMP-9 concentration [53]. At the same time, TNF-α decreases MMP-2 concentration and activity as well as hCG secretion [53,84]. It is therefore suggested, that high TNF-α levels presented during inflammatory responses [85], could be responsible in pathologic processes such as pregnancy loss, preterm delivery and preeclampsia, for abnormal trophoblast endocrine function [84,86]. In a recent study, despite increased MMP-9 expression, TNF-α was found to inhibit *in vitro *trophoblast migration and invasion. However, the plasminogen activator inhibitor-1 (PAI-1) that blocks the plasminogen activator system, was found to be increased [86]. Thus, TNF-α seems to exert diverse *in vitro *effects, depending on individual trophoblast preparation, type of cell-line and cytokine concentration. This affects the value of conclusions which can be derived from *in vitro *studies.

A link between elevated PAI-1 levels detected in plasma and syncytium of preeclamptic women, and elevated TNF-α found in preeclamptic sera, villi and deciduas has been suggested [86]. The elevated TNF-α level could result from local hypoxic conditions developing under reduced maternal and fetal vascular perfusion [87].

### 7.3 Prostaglandins

Prostaglandins are synthesized from arachidonic acid by phospholipase A_2_, followed by cyclooxygenase (COX). Prostaglandin E_2 _(PGE_2_) is essential for mammalian female reproduction. PGE_2 _is involved in regulation of decidualization of endometrial stomal cells [88], apparently through stimulation of IL-11, since blocking of IL-11 in PGE_2 _treated cells reduces decidualization and COX inhibitor reduces IL-11 secretion from these cells [89]. In rats, expression of the PGE receptor EP2 is highly detected at implantation sites in luminal epithelium, peaking on day 6 of pregnancy [90]. Phospholipase A2 and PGE_2 _receptor were described to be up-regulated in human endometrium during the window of implantation [91]. Experiments in mice have shown that prostaglandins are essential for the correct timing of implantation [92]. Reduced levels of COX-2 or COX-2 ligands cause deferred implantation and reduced litter size, and prostaglandin treatment resumes on-time implantation [92]. COX-1, COX-2 and prostaglandin E synthase (PGES), which catalyses COX products to PGE_2_, are highly expressed in mice at the blastocyst stage [93].

## 8. Extracellular degrading matrix proteinases

### 8.1 MMPs

The matrix metalloproteinases (MMPs) are a family of zinc-containing endopeptidases capable of degrading all components of the ECM, both interstitial matrix and basement membrane. MMPs are thought to play an important role in tumor progression and metastasis [94]. Twenty-six mammalian and twenty-two human MMPs are known so far [28,95]. The vertebrate MMPs each have distinct but often overlapping substrate specificities. Human MMPs fall into five classes according to primary structure and substrate specificity: collagenases, gelatinases, stromelysins, membrane-type and nonclassified MMPs [96]. MMPs regulate cell behavior in numerous ways, including cell-matrix and cell-cell interactions and the release, activation or inactivation of autocrine or paracrine signaling molecules and cell surface receptors. ECM-degradation permits cellular invasion to take place and influence processes such as cell shape, movement, cytoskeleton machinery and matrix-derived signals [95]. MMPs are regulated at several levels, including transcriptional, secretion, activation, inhibition and degradation. Transcriptional regulation is cell, tissue and MMP-specific and includes several cytokines and growth factors [97]. MMPs are produced as proenzymes, requiring removal of the propeptide domain for activation. The extracellular activation of most MMPs can be initiated by activated MMPs or by several serine proteinases, including uPA, plasminogen, thrombin and elastase. MMP-2 is activated at the cell surface through a multi-step pathway involving membrane type (MT) – MMPs and TIMP-2 [98]. MMPs are inhibited by α2-macroglobulin, in tissue fluids, and in tissue by TIMPs, which bind to MMPs in a 1:1 stoichiometric fashion [99].

*In vitro *studies suggest that successful implantation and placentation result from the balance between secretion of MMPs from the trophoblast and their inhibition by TIMPs [100,101]. The gelatinases, MMP-2 and -9, degrade Collagen IV, the main component of the basement membrane, and are therefore regarded as key enzymes in the implantation process, enabling the invasion of the trophoblast cells through the decidua and into the maternal vasculature. Several *in vitro *studies have found the gelatinases to be required for trophoblast invasion [102-105,32].

Gelatinases, mainly MMP-2, are secreted from the embryo already at the blastocyst stage [106-108]. Lately we and others have shown, that MMP-2 and MMP-9 have a differential expression throughout the first trimester, with MMP-2 being the main gelatinase in early first trimester (6–8 w) and MMP-9 being dominant in late first trimester (9–12 w) [32,104]. MMPs may have other important actions in the implantation process, besides ECM degradation, including regulation of bioactivity of growth factors, cytokines and angiogenic factors [27]. This includes MMP-2 or -9 activation of TGFβ [109] or release of IGF by degradation of IGFBPs [110]. Another role may be modulation of angiogenic factors such as endothelin-1, a vasoconstrictor [111], or angiostatin, an angiogenic inhibitor [112]. Interestingly, knockout mice, deficient in MMP-2 or MMP-9, are fertile and only mild effects have been reported: MMP-2 deficient mice have a subtle delay in their growth [113] and MMP-9 deficient mice show a decreased litter size and an increase in percentage of infertile mice [114].

MT1-MMP deficient mice die before puberty; therefore no conclusions can be made on reproductive capacity [115]. In MMP-7 null-mice both MMP-3 and MMP-10 were up-regulated in the uterus, whereas in MMP-3 deficient mice MMP-7 and MMP-11 were up-regulated, thereby indicating the presence of a compensation mechanism [116].

The MMP gene promoter contains several *cis*-regulatory elements, often acting synergistically, with varying importance and effect, depending upon cell-type and inducer. AP-1 sites give several MMP genes in various cell types the ability to be induced by phorbol esthers and act in some cases synergistically with adjacent Ets-binding sites [117,2,118]. AP-1 was found to be necessary, but not sufficient for transactivation of the MMP-9 gene in human trophoblasts, and antisense against Jun and Fos transcription factors, belonging to the AP-1 complex, was found to inhibit MMP-9 gelatinolytic activity in trophoblasts [118]. The importance of the Ets site was shown by the study of knockout mice for ets2 transcription factor, which resulted in deficient MMP-9 expression and early embryonic lethality [119]. Lately, it has been shown that EGF induction of MMP-9 as well as TIMP-1 in an extravillous trophoblastic cell-line is through the MAPK and PI3K pathways [37].

Recently ADAM (a disintegrin and metalloproteinase, adamalysin)-19 has been detected during early pregnancy in the endometrium and the placenta of the rhesus monkey, and may therefore also be involved in trophoblast invasion and degradation of the ECM [120].

### 8.2 TIMPs

Tissue inhibitors of matrix metalloproteinases (TIMPs) are the main inhibitors of MMPs in tissue, physiologically controlling their activity. The four known TIMP proteins (TIMP 1–4) inhibit MMPs in a 1:1 stoichiometric fashion, by interaction of mainly the C-terminal with the MMP catalytic site. Individual TIMPs differ in their ability to inhibit various MMPs, and in their gene regulation and tissue-specific patterns of gene expression [121]. TIMP-1 and 2 exhibit inhibitory activity against the active forms of all MMPs, TIMP-1 preferentially binding MMP-9 in both active and latent form [122], and TIMP-2 preferentially binding active or latent MMP-2 [123]. TIMP-2 in addition has an important role in activation of MMP-2, together with MT1-MMP.

TIMPs (1–3) are produced by trophoblastic and decidual tissues throughout gestation [124,125,100]. TIMP-4 is secreted from mouse blastocyst, and the addition of specific TIMP-4 antibody increases the expression and activity of MMP-2 and MMP-9 [126]. In addition this group also found TIMP-4 to be expressed in a malignant choriocarcinoma human cell-line (JEG-3) [127]. Lately TIMP-1 and -3 and to a lesser extent TIMP-2 were detected in pre-implantation human embryos, indicating that MMP and TIMP genes are among the first genes to be expressed in the developing embryo, preparing for implantation [128]. Growth factors and cytokines known to effect trophoblast invasiveness may act by up or down-regulation of TIMPs. TGF-β for example inhibits trophoblast invasion by up-regulating TIMP-1 and PAI-1 and down-regulating uPA [129]. TIMPs may have additional roles besides MMP inhibition, including increasing cell proliferation [130,131] and embryo development [132].

### 8.3 Serine proteases

The plasminogen activator system includes the urokinase-type plasminogen activator (uPA), the tissue-type plasminogen activator (tPA), the PA inhibitors PAI-1 and PAI-2 and the cell surface uPA receptor. The PA system converts plasminogen into the active serine protease plasmin, which can degrade ECM. The activity of the PA system is balanced by the inhibitors (PAI-1 and -2) [133]. Besides directly degrading ECM, the PA system has an indirect effect, through proteolytic activation of MMPs. Both uPA and plasmin are reported in the uterus [134] and in trophoblasts [135]. Studies in mice and rats suggest a role for the PA system in the implantation process [136]. A recent report found that Adrenomedullin (ADM), a polypeptide belonging to calcitonin gene-related peptide superfamily, enhances *in vitro *trophoblast proliferation and invasion [137]. Both ADM binding sites and ADM are present in the trophoblasts, the latter is most abundant in first trimester placenta [138]. The report showed that ADM decreased PAI-1 expression and increased MMP-2 activity, thereby enhancing the downstream reaction of cell invasion [137].

## 9. Endovascular invasion

The development of a placental vascular network is essential for the growth and maintenance of the developing embryo. Several factors are involved in this angiogenic process, including VEGF (vascular endothelial growth factor), PDGF (platelet-derived growth factor) and PAF (platelet-activating factor) [26].

VEGF induces angiogenesis and increases permeabilization of blood vessels. VEGF and its receptors are expressed in both the endometrium and in trophoblastic cells [1]. Mouse embryos with functional inactivation of one VEGF allele die on day 11–12 of pregnancy and show several malfunctions on the vascular system [139]. VEGF mRNA expression can be detected already in the blastocyst, enabling the implanting embryo to induce angiogenesis at the implantation site by binding to endometrial receptors [1]. VEGF expression is up-regulated in placental tissues by hypoxia, associated with early placental development, whereas another angiogenic factor PIGF (Placental growth factor) is down-regulated [140]. Surprisingly placental VEGF was reduced in pre-eclamptic pregnancies, despite the prolonged hypoxic condition associated with pre-eclampsia [141]. PIGF, on the other hand, was decreased in pre-eclampsia, as expected [142]. TGF-β and TNF-α, two pro-angiogenic factors present in the uterus, increased VEGF expression in a trophoblast cell-line [143]. This is especially interesting in light of the elevated TGF-β level, thought to be involved in pre-eclampsia [42], which may be an attempt to raise the low VEGF level.

ICAM (intercellular adhesion molecule), VCAM (vascular cell adhesion molecule) and PECAM (platelet endothelial cell adhesion molecule) are endothelial-cell adhesion molecules, playing an important role in endothelial activation and are elevated in the maternal circulation during pregnancy. Co-culture of endothelial cells and trophoblasts lead to an increased expression of ICAM, VCAM and E-selectin, indicating that factors released from the trophoblasts activate endothelial cells [108]. In pre-eclamptic pregnancies the expression of these adhesion molecules in the maternal circulation is further increased [108,144]. Elevated expression of E-selectin, as found in pre-eclampsia and in placental tissues cultured under hypoxia-reoxygenation conditions, may be mediated by the cytokine tumor necrosis factor-alpha, since anti-TNFα antibody reduced this activation [145]. Elevated VCAM in maternal blood together with elevated hyperhomocyst(e)inemia, a preeclampsia risk factor, were found to be strongly associated with an increased risk of preeclampsia [146]. This is in contrast to the result of an immunocytochemical study of placental bed biopsies from normal and pre-eclamptic women, which found no difference in ICAM, PECAM, VCAM and E-selectin expression between the two groups, suggesting that these molecules are not implicated in the etiology of pre-eclampsia [147]. In another study MCAM (melanoma cell adhesion molecule) expression was reduced in trophoblasts in placentas from pre-eclamptic women compared to normal, as detected by immunohistochemistry [148]. VCAM is lower in term than in first trimester placenta, indicating importance in the developmental stage. The reduced VCAM expression found in pregnancies complicated with fetal growth restriction further supports this concept [149]. In a comparison study of first trimester serum between normal pregnant women and women with pregnancy-induced hypertension (PIH) ICAM and E-selectin, but not VCAM or P-selectin, were significant higher in women who developed PIH late in gestation, suggesting that these factors can serve as effective indicators of the onset of PIH [150].

## 10. Conclusion

Ethical restrictions and limited availability of human tissue confined our studies on human implantation. It must be appreciated that most knowledge comes from *in vitro *experiments using cultured human trophoblasts or cell-lines and *in vivo *studies from other species. All the same, it is clear today that the implantation process depends on appropriate timing and is regulated by various factors of both maternal and embryonic origin. The success of this process is a result of complex interactions between these factors and comprehension of the process demands further characterization of these interactions.

New technologies that allow the profiling of tissues at the genomic, transcriptomic and proteomic levels are becoming available will probably bring more information and hopefully will help to shed more light on the implantation process. Further understanding of the process will enable new strategies in treating implantation failure both in natural and in assisted reproduction.
